# Risk-Based Prioritization among Air Pollution Control Strategies in the Yangtze River Delta, China

**DOI:** 10.1289/ehp.1001991

**Published:** 2010-05-17

**Authors:** Ying Zhou, Joshua S. Fu, Guoshun Zhuang, Jonathan I. Levy

**Affiliations:** 1 Department of Environmental and Occupational Health, Rollins School of Public Health, Emory University, Atlanta, Georgia, USA; 2 Department of Environmental Health, Harvard School of Public Health, Boston, Massachusetts, USA; 3 Department of Civil and Environmental Engineering, University of Tennessee, Knoxville, Tennessee, USA; 4 Department of Environmental Science and Engineering, Fudan University, Shanghai, People’s Republic of China

**Keywords:** air pollution, China, CMAQ, health risk, ozone, PM_2.5_, Yangtze River Delta

## Abstract

**Background:**

The Yangtze River Delta (YRD) in China is a densely populated region with recent dramatic increases in energy consumption and atmospheric emissions.

**Objectives:**

We studied how different emission sectors influence population exposures and the corresponding health risks, to inform air pollution control strategy design.

**Methods:**

We applied the Community Multiscale Air Quality (CMAQ) Modeling System to model the marginal contribution to baseline concentrations from different sectors. We focused on nitrogen oxide (NO_x_) control while considering other pollutants that affect fine particulate matter [aerodynamic diameter ≤ 2.5 μm (PM_2.5_)] and ozone concentrations. We developed concentration–response (C-R) functions for PM_2.5_ and ozone mortality for China to evaluate the anticipated health benefits.

**Results:**

In the YRD, health benefits per ton of emission reductions varied significantly across pollutants, with reductions of primary PM_2.5_ from the industry sector and mobile sources showing the greatest benefits of 0.1 fewer deaths per year per ton of emission reduction. Combining estimates of health benefits per ton with potential emission reductions, the greatest mortality reduction of 12,000 fewer deaths per year [95% confidence interval (CI), 1,200–24,000] was associated with controlling primary PM_2.5_ emissions from the industry sector and reducing sulfur dioxide (SO_2_) from the power sector, respectively. Benefits were lower for reducing NO_x_ emissions given lower consequent reductions in the formation of secondary PM_2.5_ (compared with SO_2_) and increases in ozone concentrations that would result in the YRD.

**Conclusions:**

Although uncertainties related to C-R functions are significant, the estimated health benefits of emission reductions in the YRD are substantial, especially for sectors and pollutants with both higher health benefits per unit emission reductions and large potential for emission reductions.

The Yangtze River Delta (YRD), which generally refers to southern Jiangsu Province, eastern and northern Zhejiang Province, and the municipality of Shanghai, is the fastest growing economic development region in China and one of the most densely populated regions in the world. Shanghai is one of the world’s largest cities, with > 18 million long-term residents and a population density of > 40,000 people/km^2^ in some districts. Accompanying this economic development has been a dramatic increase in energy consumption and air pollution emissions. For example, although the Shanghai metropolitan area and the provinces of Jiangsu and Zhejiang constitute only 2% of the area of China, their emissions of sulfur dioxide (SO_2_), nitrogen oxides (NO_x_), and fine particulate matter [aerodynamic diameter ≤ 2.5 μm (PM_2.5_)] accounted for 12%, 15%, and 12%, respectively, of total emissions in China in 2006, which increased by 36%, 55%, and 14%, respectively, from 2001 to 2006 (Zhang et al. 2009). NO_x_ emissions are of particular concern because they have increased the fastest and are forecasted to increase even more (Chen et al. 2006).

Several studies (Kan et al. 2004; Li et al. 2004; Streets et al. 1999) have evaluated the health benefits of air pollution control in Shanghai, primarily SO_2_ and PM_10_, and occasionally sulfate particles. Similar studies have been conducted in other parts of China, such as a recent estimate of annual deaths attributable to air pollution in the Pearl River Delta (Loh et al. 2008). In another study in the Pearl River Delta area, Wang et al. (2005) investigated how the emissions from different sectors influenced the concentrations of gaseous pollutants including ozone. And Wang and Mauzerall (2006) quantified the total health damages from PM due to anthropogenic emissions from Zaozhuang, Shangdong Province. Besides these studies on regional air pollution, both the magnitude of the air pollution problem in China at the national level and the contribution from the power plants have been estimated by several studies, including one of the first to quantify the national burden of air pollution (World Bank 1997). In another study, Wang and Smith (1999) focused on the electric power sector in the context of determining the secondary benefits of greenhouse gas reductions. In addition, a large-scale study conducted (Ho and Nielsen 2007) assessed the health damages of air pollution in China and examined several pollution control policies and how they might affect economic performance.

Researchers have pointed out that emission reductions in different sectors may have different levels of effectiveness on reducing human exposure (Li et al. 2004; Streets et al. 1999) and that the benefits of many pollution control measures likely far exceed the costs; however, the variance by sector and its policy implications for future pollution control have not been investigated systematically in China. For example, none of the previous studies included both ozone and PM_2.5_ in assessing the health damages, although exposures have been associated with increased mortality and a variety of other health outcomes (Bell et al. 2006, 2007; Levy et al. 2005; Pope and Dockery 2006). The exclusion of ozone is partly because of past emphasis on the power sector, where SO_2_ and PM receive greater attention, but is also attributable to limitations in the atmospheric models used in previous studies (Li et al. 2004). Also, most studies used PM_10_ and total suspended particles (TSPs) to estimate population exposure to PM in China, whereas epidemiologic studies in the United States and worldwide have demonstrated more robust associations with PM_2.5_.

Our study will fill this gap by comparing how emission control strategies across different sectors (e.g., power generation, mobile sources, industry) influence population exposures and health risks related to PM_2.5_ and ozone in the YRD area. The sectoral details will help guide development strategies that are economically and environmentally optimal, providing the basis for policy makers to determine how to prioritize future control efforts among the different sectors in the YRD.

## Materials and Methods

In this study, we applied air pollution health impact assessment methods, following approaches articulated elsewhere (National Research Council 2002; World Health Organization 2000). Briefly, this entailed estimating baseline emissions and the marginal contribution from individual source categories, use of a chemistry-transport model to characterize population exposures associated with source category emissions, and application of concentration–response (C-R) functions from the epidemiological literature along with characterization of population patterns to quantify health impacts.

### CMAQ modeling and emission inventory

A state-of-the-science Eulerian grid model—the Community Multiscale Air Quality (CMAQ) modeling system [version 4.6; U.S. Environmental Protection Agency (EPA), Research Triangle Park, NC, USA] was applied to an emission inventory we developed to estimate the baseline concentrations as well as the marginal concentration change associated with hypothetical control strategies for multiple sectors in the YRD. CMAQ has capabilities to simulate the various chemical and physical processes important for understanding atmospheric processes and thus allowed us to model population exposure to pollutants such as ozone and PM_2.5_. Although CMAQ is currently used in analyses in the United States (Cohan et al. 2007) and in some air quality assessments in China (Fu et al. 2009), it has rarely been used for risk assessments in China and other developing countries. We applied a three-way nested model with 27-km (covering all of China), 9-km (covering eastern China), and 3-km (covering the YRD) grid resolutions, respectively [see Supplemental Material, Figure S1 (doi:10.1289/ehp.1001991)]. Outputs from the 27-km and 9-km domains were used as boundary conditions for the 3-km domain. To develop meteorological inputs for CMAQ, we used the fifth-generation mesoscale model (MM5) developed by the U.S. National Center for Atmospheric Research. For more detailed information on CMAQ and MM5 configurations, see Supplemental Material, Table S1.

The emission inventory for the YRD consisted of emissions mainly from four sectors: power plants, industrial sources (e.g., metallurgical, mineral, cement, chemical industries, small industries such as coke and brick production), mobile sources, and domestic life (e.g., livestock, residential, and biomass burning). Power plants and emissions from production processes of major industrial sources were modeled as point sources. Mobile sources mainly included on-road vehicle emissions in the major cities. Emissions from domestic life and the fugitive emissions from industry were modeled as area sources.

In developing our emission inventory, we used the Asian emission inventory for 2006 that was developed for the INTEX-B project of the U.S. National Aeronautics and Space Administration (Zhang et al. 2009). The resolution of the INTEX-B emission inventory is 30 min × 30 min (~ 55 km × ~ 55 km). To capture finer resolution within our emission inventory, we incorporated various sources of input, using collaborations with local agencies, Google Earth (version 5.1.3535.3218; Google Inc., Mountain View, CA, USA) to identify the locations of large point sources based on their addresses, updated road networks and LandScan population data to characterize the distribution of mobile source emissions, and the statistical yearbook of the major cities in the YRD for domestic fuel emissions from the consumption of coal, liquified petroleum gas, coal gas, and natural gas.

After conducting the base–case simulation using CMAQ, we validated it using available monitoring data for ozone and PM_2.5_ collected in Shanghai before proceeding with additional model runs. Because PM_2.5_ is not currently a criteria air pollutant in China and there are no publicly available ozone monitoring data, the monitoring data we had access to for model validation were all from research monitoring sites located in Shanghai and operated by Fudan University [see Supplemental Material, Figure S2 (doi:10.1289/ehp.1001991)]. Hourly ozone monitoring data were available from three stations for 4 weeks (2–9 May, 12–19 June, 10–17 July, and 14–21 August 2006). Daily average PM_2.5_ monitoring data were available from one station for 1–29 April, 1–26 August, and 1–28 November 2006.

Once the validation of the baseline run was satisfactory, we designed scenarios to model the marginal contribution of selected emission sectors in the YRD to the base-line concentrations in the 3-km resolution domain. Although some underestimation of the total health benefits would have resulted, given the significant regional transport of PM_2.5_ and ozone, the size of the 3-km domain provides YRD environmental authorities with the most relevant information for control strategy prioritization and decision making.

For this study, we modeled sectoral impacts in total (e.g., all power plants in the YRD simultaneously). Table 1 lists the emission reduction scenarios in more detail. In designing the scenarios, we considered several factors. First, we focused on NO_x_ emission control, a stated interest of decision makers and a pollutant for which emissions have been increasing the fastest in recent years among the pollutants modeled (Zhang et al. 2009). Second, because NO_x_ emissions influence PM_2.5_ and ozone concentrations, and given the anticipated health impacts of PM_2.5_ and ozone, we also considered other pollutants [e.g., SO_2_, volatile organic compounds (VOCs), and primary PM_2.5_] that can affect PM_2.5_ and ozone concentrations. Lastly, we tried to cover the current and proposed pollution control measures by the Shanghai government in preparation for World Expo 2010 (Chen CH, personal communication), although these scenarios do not necessarily correspond to specific official control measures. In each of the four major sectors (power, industry, mobile, and domestic), we included scenarios for reducing NO_x_ alone (scenarios 1, 3, 7, and 10) and scenarios for reducing NO_x_ along with other pollutants to evaluate the relative magnitude of impacts (scenarios 2, 5, and 9). We also considered scenarios for reducing VOC alone (scenarios 4 and 8), allowing for analyses of interactions between NO_x_ and VOC controls, as well as one scenario (scenario 6) to check for non-linearity of concentration changes to the magnitude of emission reductions. The magnitude of the emission reductions corresponds with specific control technologies for the power sector and an approximation of technologically and financially plausible emission reductions in other sectors, although the logistical feasibility and costs of controls clearly vary across sectors.

In each case, we estimated the total exposure and public health impacts but focused on the marginal impacts per ton of emissions, allowing for direct comparisons among sectors. Although CMAQ calculates speciated PM_2.5_ concentrations, we report the total PM_2.5_ concentrations in calculating the population exposure under different scenarios, which includes changes in multiple constituents. To estimate the pollutant-specific benefits, we used the incremental population exposure change between scenarios. For example, the difference in population exposure between scenario 1 and the base–case allowed us to calculate exposure reductions associated with NO_x_ emissions, whereas the difference between scenarios 2 and 1 allowed us to approximate the exposure reductions associated with SO_2_ emissions.

### Population data

To calculate the population exposure and subsequent health impacts, we collected population data from two different sources. First, we used LandScan 2007 as the primary basis for estimating exposures. LandScan is a worldwide population database compiled on a 30-second × 30-second latitude/longitude grid. Census counts (at subnational level) were apportioned to each grid cell based on likelihood coefficients, which are based on proximity to roads, slope, land cover, night-time lights, and other information (Dobson et al. 2000; Oak Ridge National Laboratory 2009). Similar to Census data, LandScan provides a single population estimate for each location, although these estimates include diurnal movements and collective travel habits, whereas most censuses count people at their night-time residences. In addition, LandScan includes annual updating of data inputs, as well as global coverage, which potentially allows for easy comparisons with other parts of the world in the future.

However, LandScan does not provide the same level of detail as Census data in terms of population demographics, so we obtained additional information necessary for sub-sequent calculation of health benefits (e.g., baseline mortality rates) from China Census 2000 (China Data Center 2003). To calculate the health benefits, we applied spatially variable baseline mortality rates, to reflect the fact that the YRD area includes both urban and rural areas and that their residents may have different disease patterns, socioeconomic status, and life expectancy. For counties in the YRD, the average baseline mortality rate is 0.6%, with the 5th and 95th percentile rate at 0.34% and 0.84%, respectively. We assumed these estimates based on Census 2000 are applicable to population estimates from LandScan 2007 data.

### Population exposure

The population exposure change under different scenarios was calculated by combining population in each location with the corresponding concentration change. Geographical information system (ArcGIS) software (version 9.3; ESRI, Redlands, CA, USA) was used to convert population data to match the grid structure of CMAQ. Because the emissions of different pollutants under study vary significantly by sector, we focused our analyses on the marginal benefits per ton of emission reductions. We facilitated these comparisons by using the concept of intake fraction (iF), the fraction of a material released from a source that is inhaled or ingested (Bennett et al. 2002). We calculated iF as (∑ *C_i_* × *P_i_*) × (*BR*/*Q*), where *C_i_* is the marginal concentration in grid cell *i* (micrograms per cubic meter) associated with source emission rate *Q* (micrograms per day, noting that *Q* can be the same pollutant as *C* or a precursor to *C*), *P_i_* is the population count in the grid cell, and *BR* is a nominal breathing rate of 20 m^3^/day. We calculated iFs for primary PM as well as secondary PM associated with various particle precursors (SO_2_, NO_x_, VOCs) and ozone (defined as the mass of ozone inhaled per unit mass of NO_x_ or VOC emissions). Because numerous particle constituents are influenced by precursor emission changes in CMAQ, we did not focus on iF values for individual constituents but discuss the dominant constituents for all secondary PM iFs. In addition, because emissions of multiple pollutants can influence PM_2.5_ and ozone concentrations, we estimated the pollutant-specific and sector-specific iFs by comparing the population exposures among scenarios.

### Health effects

Although detailed characterization of health risks is beyond the scope of our investigation, we developed C-R functions for PM_2.5_ and ozone mortality to allow our CMAQ outputs to be integrated into a common metric and to allow for initial evaluation of the magnitude of health benefits, the dominant pollutants, and the key uncertainties. For both PM_2.5_ and ozone, we determined a central estimate, plausible lower bound, and plausible upper bound. These are not meant as formal 95% CIs but were used to construct uncertainty distributions when combining PM_2.5_ and ozone health benefit estimates.

As a general point, there are multiple limitations in applying C-R functions largely derived from the United States or Europe to China. There are differences in baseline disease patterns and age distributions, health care systems, pollutant levels and composition, and exposure modifiers. There are also complexities given the more recent focus on PM_2.5_ in U.S. and European studies but the use of PM_10_ or TSP in China given available monitoring data. To develop applicable C-R functions, we used a combination of evidence from the global literature and the Chinese literature.

First we considered PM: Two studies that developed C-R functions applicable to China (Aunan and Pan 2004; Levy and Greco 2007) concluded that the Chinese time-series mortality literature yielded estimates on the order of 0.3–0.4% increases in all-cause mortality per 10-μg/m^3^ increase in daily PM_10_ concentrations, slightly lower than the global literature. In a recent study that examined three cities in China (Wuhan, Shanghai, and Hong Kong), Wong et al. (2008) found a pooled C-R function for time-series mortality of 0.37% (95% CI, 0.21–0.54%), similar to the values reported above. C-R functions in China were higher for cardiovascular and respiratory mortality, with patterns similar to those seen in the global literature.

However, health risk assessments for PM generally apply evidence from cohort mortality studies (National Research Council 2002; World Health Organization 2000), given the strength of available studies and supporting evidence for mortality risks from long-term exposure (e.g., evidence that PM contributes to accelerated atherosclerosis; Floyd et al. 2009). Recent syntheses of the cohort mortality literature (Levy et al. 2009) and expert elicitation studies (Industrial Economics 2006) found that a 1% increase in mortality per 1-μg/m^3^ increase in annual PM_2.5_ concentrations was a reasonable central estimate, falling between estimates from the Harvard Six Cities Study (Laden et al. 2006; Schwartz et al. 2008) and the American Cancer Society study (Jerrett et al. 2009; Pope et al. 2002). There is no cohort mortality evidence available in China, but earlier cross-sectional studies yielded C-R functions roughly comparable to those from the U.S. cohort studies (Levy and Greco 2007).

Despite the lack of Chinese cohort mortality evidence, the literature is sufficiently compelling to indicate that mortality risk due to long-term exposure would be expected, and comparisons of the time-series estimates indicate reasonable concordance between the Chinese and U.S. literature despite the large differences in ambient concentrations and other factors. Thus, for our central estimate, we used a 1% increase in all-cause mortality per 1-μg/m^3^ increase in annual PM_2.5_ concentrations. For our bounds, we note that a recent study (Levy et al. 2009) used 0.3% as a lower bound and 2.0% as an upper bound, representing the median values across experts for the 5th and 95th percentiles of the uncertainty distribution in the recent expert elicitation study. We maintained this upper bound (which slightly exceeds the central estimate from the Harvard Six Cities Study) but used a lower bound of 0.1%, reflecting a value similar to the time-series evidence and the uncertainties in determining a cohort mortality effect in China without direct evidence. These C-R functions were applied identically to all particle constituents, given a lack of systematic information to support quantitative differential toxicity, especially with respect to atmospheric conditions in China.

Ozone had not been characterized in previous studies developing C-R functions for China, in part because of a lack of systematic evidence in the global literature at the time of those investigations. However, three recent meta-analyses and multicity studies (Bell et al. 2005; Ito et al. 2005; Levy et al. 2005) found evidence of an independent ozone effect in the time-series literature; ozone mortality was recently evaluated in multiple Chinese cities (Wong et al. 2008), and recent evidence from the American Cancer Society cohort study (Jerrett et al. 2009) provides some indication of a long-term ozone effect on respiratory mortality. Thus, ozone mortality merits inclusion in our investigation.

Using standard units conversions and an assumption of an approximate 3:4 ratio between 8-hr maximum and 1-hr maximum concentrations (Levy et al. 2005), the three meta-analyses (Bell et al. 2005; Ito et al. 2005; Levy et al. 2005) yield C-R functions for all-cause mortality of 0.21% (95% CI, 0.15–0.32%), 0.27% (95% CI, 0.18–0.35%), and 0.28% (95% CI, 0.21–0.35%), respectively, per 10-μg/m^3^ increase in 8-hr maximum ozone. The recent multicity study in Asia (Wong et al. 2008) found that, for the three Chinese cities combined, the C-R function for all-cause mortality was 0.31% (95% CI, 0.13–0.48%). Central estimates were very similar for cardiovascular and respiratory mortality (0.29% and 0.23%, respectively, although both estimates lacked statistical significance), and the all-cause mortality estimate for Shanghai is identical to the three-city pooled estimate. As for PM, little evidence exists that the ozone time-series mortality C-R function differs systematically between the Chinese cities and the global literature.

Similar to PM_2.5_, there is no evidence of ozone cohort mortality for China. In the U.S. cohort literature, an ozone effect on mortality was significant in one recent publication (Jerrett et al. 2009), but only for respiratory mortality in models including PM_2.5_. The C-R function for respiratory mortality corresponded to a 2.7% increase per 10-μg/m^3^ increase in 8-hr maximum ozone following the conversions above. This is significantly greater than the time-series estimates (albeit for respiratory mortality, only a fraction of all-cause mortality), but because of the lack of an impact for all-cause mortality or corroboration from other studies, we do not use this evidence for our primary C-R functions.

Combining this evidence, we considered 0.3% reduction in all-cause mortality per 10-μg/m^3^ reduction in 8-hr maximum ozone as a reasonable central estimate (reflecting the Chinese three-city study and two of the meta-analyses), with 0.15% as a lower bound (reflecting the lower confidence limits of the various studies) and 0.45% as an upper bound (reflecting the upper confidence limit of the Chinese three-city study as well as modest weight on the emerging cohort mortality evidence).

### Health benefit estimation

Although we did not develop the upper and lower bound C-R functions as specific percentiles of uncertainty distributions, we wished to estimate net health benefits across PM_2.5_ and ozone, necessitating some combination of distributions. To approximate the overall net mortality change considering the uncertainty in C-R functions, we assumed that the C-R functions for PM_2.5_ and ozone each follows a triangular distribution with central estimate as the mode and the lower and upper bounds as the minimum and maximum values for the distribution. We performed Monte Carlo analysis using SAS 9.1 (SAS Institute Inc., Cary, NC, USA) to combine the distributions, noting that uncertainty in other risk assessment components was not considered.

## Results

### Comparison between CMAQ modeling and monitoring data

To validate the performance of CMAQ, we compared ozone and PM_2.5_ modeling results in the base–case scenario with observation data for part of 2006 at four monitoring stations in Shanghai, using U.S. EPA guidance (U.S. EPA 2007). The mean normalized bias (MNB) between model and observational data was relatively low for both daily average PM_2.5_ (0.5%) and daily maximum 8-hr ozone (−4.3%), indicating a lack of systematic model bias. For hourly ozone, the model underestimated concentrations somewhat, with MNB of −25.3% and normalized mean error of 29%. In general, model performance was considered adequate for our application.

### Comparison between census and LandScan data

To provide validation of population counts, we compared China Census 2000 and LandScan 2007. For the 3-km resolution YRD domain, the mean percentage difference by county was about 9%. Supplemental Material, Figure S3 (doi:10.1289/ehp.1001991) shows the number of people in each grid cell in the YRD based on LandScan 2007 data. There was an average of 6,407 people in each grid cell with the maximum of 669,239 (in the Shanghai metropolitan area), corresponding to population densities of 712 and 74,360 people/km^2^, respectively.

### Base–case emissions and ambient concentrations

In Table 2, we show the estimated emission rates by sector and pollutant in the base–case scenario. Of note, emissions of many pollutants are high in the industry sector, in part due to the active manufacturing industry in the YRD and the lower penetration rate for pollution control technologies compared with the power sector.

**Figure 1:**
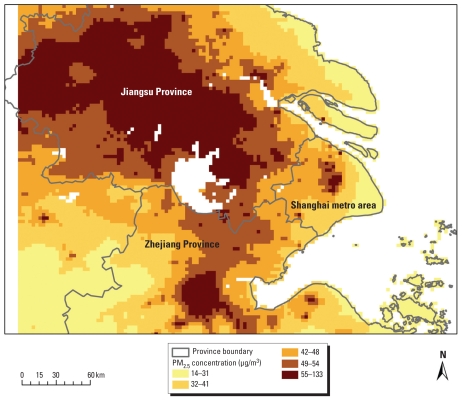
Estimated annual average PM_2.5_ concentration (μg/m^3^) in the YRD domain in the base–case scenario.

Figure 1 shows the annual average PM_2.5_ concentration in the YRD domain in the base–case scenario. The estimated mean annual PM_2.5_ concentration in the YRD domain was 38.4 μg/m^3^, although with significant variation across the study domain (range, 12.7–132.8 μg/m^3^). Similarly, Figure S4 in Supplemental Material (doi:10.1289/ehp.1001991) shows the annual average 8-hr maximum ozone concentration, which ranged from 17 to 54 ppb.

### iF variation by pollutant and sector

To estimate the pollutant-specific iFs, we compared the population exposures among scenarios (Table 3). iFs ranged significantly across pollutants, with more modest differences across sectors. Primary PM_2.5_ from the industry and mobile source sectors have the highest iF of 1.4 × 10^−5^, which means that for every metric ton of primary PM_2.5_ emitted from either sector, 14 g is eventually inhaled by the total population in the YRD domain. For secondary PM_2.5_, the iF was greatest for SO_2_ emissions from the power sector, with a value of 1.2 × 10^−6^. Among the different species of PM_2.5_ modeled by CMAQ, 98% of the PM_2.5_ concentration reduction was attributable to sulfate and ammonium particles. For secondary PM_2.5_ from NO_x_ emissions, the iFs from power plants, mobile sources, and industry are nearly identical (~ 3.9 × 10^−7^). In each case, the concentration reduction was driven by nitrate and ammonium reductions with an offsetting increase in sulfate (35% of the magnitude of the nitrate and ammonium reductions for the power sector, 20% for mobile sources, and 19% for industry). In contrast, the domestic emissions sector has a negative iF for secondary PM_2.5_ from NO_x_ emissions, potentially attributable to two factors. The low NO_x_ emissions within the domestic sector translated into a small reduction in secondary nitrate population exposure. Second, when compared with other scenarios, the domestic sector had the greatest increase in ozone concentrations per unit NO_x_ emission reductions, which contributed to greater oxidizing power of the atmospheric and subsequent increases in other PM_2.5_ species (e.g., secondary sulfate and secondary organic aerosols). As a result, there is an increase in overall PM_2.5_ concentration corresponding to the NO_x_ emission reductions within the domestic sector.

Across all scenarios, reducing NO_x_ emissions alone led to an increase in ozone population exposure (as indicated by the negative values in Table 3, “Ozone from NO_x_”). However, when VOC emissions are reduced alone, ozone population exposures are reduced and the iF is on the order of 1 × 10^−6^ (as shown in Table 3, “Ozone from VOC”). This indicates that the YRD model domain is VOC limited in terms of ozone formation, explainable by the high baseline NO_x_ emissions and low biogenic VOCs. Findings were similar using 1-hr maximum ozone concentrations, with iFs approximately 10–30% higher. Of note, within scenarios with concurrent NO_x_ and VOC controls, we observed only modest differences from the sum of NO_x_ and VOC controls applied separately.

### Health benefit variation by pollutant and sector

Although the health benefits per ton of emission reductions are approximately proportional to the iF values in Table 3 (when baseline mortality rates are about constant in different parts of the domain), the absolute benefits of the control scenarios will also depend on the magnitude of emission reductions. As indicated in Table 4, the greatest mortality reduction is achieved by controlling primary PM_2.5_ emissions from the industry sector and by controlling SO_2_ emissions from the power sector, with approximately 12,000 fewer deaths per year (lower bound of 1,200, upper bound of 24,000). This is attributable to the high primary PM_2.5_ iF and relatively high magnitude of emission reductions from the industry sector, whereas the SO_2_ emissions from the power sector have an order of magnitude lower iF but an order of magnitude higher emission reduction.

For control scenarios addressing NO_x_ emissions, the health benefits from secondary PM_2.5_ reductions (which are themselves reduced by offsetting increases in sulfate concentrations) are blunted by adverse health impacts associated with ozone increases (Table 4). Although the NO_x_ control scenarios still have positive net benefits (excluding the domestic sector), the net benefits are small relative to the aforementioned benefits for primary PM_2.5_ and SO_2_ controls.

## Discussion

To maximize the health benefits of emission reductions among different pollutants from different sectors, there are two major factors to consider—the population exposure reduction per unit emission reduction and the amount of emissions that can be reduced from different sectors. As expected and shown in prior studies in China (Zhou et al. 2003, 2006), the highest population exposure reduction per unit emission reduction for PM_2.5_ is from controlling primary PM_2.5_ emissions rather than particle precursors. Because of the relatively high primary PM_2.5_ emissions in the industry sector and the feasibility of emissions reductions, the potential health benefits are substantial.

Considering particle precursors, SO_2_ emission reductions yielded greater PM_2.5_ exposure reductions than did NO_x_ or VOC emissions reductions. In contrast, previous studies in China (Zhou et al. 2006) found similar iFs for sulfate from SO_2_ and nitrate from NO_x_, both on the order of 10^−6^. In particular, the iFs for nitrate PM_2.5_ formed from NO_x_ emissions from power plants in Shanghai and the provinces of Jiangsu and Zhejiang ranged from 2 to 5 × 10^−6^, versus 4 × 10^−7^ in the present study. The differences are likely due to two factors: The modeling domain in the previous study covers all of China, and the previous study used an atmospheric model (CALPUFF) that did not capture the offsetting increase in sulfate when NO_x_ emissions are reduced.

For ozone-related health benefits, our results show that VOC control is more effective than NO_x_ control, due to the YRD area being hydrocarbon limited in ozone formation, where ozone concentrations increase with increasing hydrocarbons (e.g., VOC) and decrease with increasing NO_x_ (Jacob 1999). One previous study (Wang et al. 2005) found that in the Pearl River Delta area of China, urban areas are VOC limited in ozone formation and the nonurban areas are NO_x_ limited, where ozone concentrations increase with increasing NO_x_ and are insensitive to hydrocarbons. Our study shows that the YRD as a whole (which contains many dense urban areas) is hydrocarbon limited, although it was beyond the scope of our study to explore the implications of source controls in different regions of the YRD or the potential long-range ozone formation that could occur from NO_x_ controls. Despite the hydrocarbonlimited ozone formation in the YRD and the relatively low particle formation per unit NO_x_ emissions, NO_x_ emission reductions in power, industry, and traffic sectors are net beneficial, across the range of C-R functions simulated for ozone and PM_2.5_.

### Limitations

Several limitations could potentially influence the interpretation of our findings. First, in estimating the health benefits per ton of emission reductions, we implicitly assumed a linear relationship between pollutant emission reductions and population exposure reductions. There are many nonlinear processes in the atmosphere chemistry that could make this assumption faulty. However, our findings suggest that nonlinearities are limited given the emission changes in our study. For example, the emission reductions in scenario 6 were 2.5 times those of scenario 5 (e.g., 50% vs. 20% reductions of NO_x_, VOC, and PM from the mobile sector), and the resulting total population exposures in scenario 6 were 2.53 times greater for PM_2.5_ and 2.44 times greater for ozone, indicating reasonable linearity.

Second, because PM_2.5_ is not currently a criteria air pollutant in China and there are no publicly available ozone monitoring data, the monitoring data we had access to for model validation are all from research monitoring sites located in Shanghai, which limited our ability to validate the model performance in other parts of the YRD modeling domain. Although further validation would have been ideal, various components of the model (e.g., the original emissions inventory input, the application of CMAQ in China) have been previously evaluated and validated, increasing our confidence in our findings. The lack of available monitoring data also emphasizes the need for publicly available comprehensive information systems in order to support health risk analyses and other environmental evaluations in China.

Third, although the C-R functions lever-aged a combination of epidemiological evidence from the global literature and from China, our health benefit estimates are dominated by risks from long-term PM_2.5_ exposure, for which there is no evidence within China. Moreover, the C-R function derived from the U.S. cohort studies would imply an extremely large mortality gradient across the YRD and between different areas of China, which is challenging to validate and interpret. That said, we did not consider it appropriate to omit cohort mortality entirely, given its biological plausibility, and no evidence exists to quantitatively deviate from the available cohort evidence. More generally, our conclusions about the relative importance of various source sectors are robust to this assumption. Our core findings therefore remain interpretable despite this large uncertainty, and we recommend that the C-R function for PM_2.5_ mortality in China be reevaluated as more evidence from China becomes available.

Fourth, our analysis considers mortality only from PM_2.5_ and ozone. Incorporation of other impacts, including morbidity outcomes or ecological damage from acid deposition, could be potentially influential if economic valuation of mortality were lower in China relative to economic valuation of morbidity than in the United States.

## Conclusion

Despite the limitations, we demonstrated in this study a systematic approach to compare the effectiveness of pollutant control strategies across different sectors in a highly exposed and highly populated region of China. The use of the state-of-the-science air quality model CMAQ and a spatially resolved emission inventory allowed us to jointly consider PM_2.5_ and ozone exposures for different emission reduction scenarios. Our findings indicate significant variation across pollutants in health benefits per ton of emission reduction. The public health benefits of realistic controls for SO_2_ emissions from the power sector and primary PM_2.5_ emissions from the industry sector are roughly comparable, given higher emission reductions for the former and higher population exposures per ton of the latter, with lower benefits from NO_x_ control strategies. This is attributable in part to the hydrocarbon-limited nature of the YRD, as well as to the lower secondary PM_2.5_ formation per ton of NO_x_ emissions relative to other particle precursors. Our findings, in combination with plausible emissions reduction estimates and their costs, provide the basis for prioritizing pollution control strategies in the YRD and provide a template for comparable analyses elsewhere.

## Figures and Tables

**Table 1:** CMAQ simulation scenarios targeting NO_x_ and other pollutant emission reductions in different sectors.

Scenario	Sector	Pollutants reduced	Reduction
1	Power	NO_x_ alone (SCR alone)	85%
2	Power	NO_x_ + SO_2_ (SCR + FGD)	85% for NO_x_ + 90% for SO_2_
3	Mobile	NO_x_ alone	20%
4	Mobile	VOC alone	20%
5	Mobile	NO_x_ + VOC + PM	20%
6	Mobile	NO_x_ + VOC + PM	50%
7	Industry	NO_x_ alone	20%
8	Industry	VOC alone	20%
9	Industry	NO_x_ + VOC + PM	20%
10	Domestic	NO_x_ alone	20%

Abbreviations: FGD, fluidized gas desulfurization; SCR, selective catalytic reduction.

**Table 2:** Estimated emission rates by sector and pollutant in the base-case scenario for the YRD domain (thousands of tons per year).

Pollutant	Industry	Power plant	Domestic	Mobile
NO_x_	479	714	73	415
VOC	1,492	198	1,121	1,019
SO_2_	816	1,464	71	11
Primary PM_2.5_	571	115	231	30

**Table 3:** iFs for primary PM_2.5_, secondary PM_2.5_, and 8-hr maximum ozone by emissions sector.

	Power		Mobile		Industry		Domestic	
Sector	iF	Scenarios compared	iF	Scenarios compared	iF	Scenarios compared	iF	Scenarios compared
Primary PM_2.5_			1.4 × 10^−5^	5, 4, 3	1.4 × 10^−5^	9, 8, 7		
Secondary PM_2.5_								
From SO_2_	1.2 × 10^−6^	2, 1						
From NO_x_	3.9 × 10^−7^	1	3.9 × 10^−7^	3	3.9 × 10^−7^	7	−2.1 × 10^−7^	10
From VOC			2.4 × 10^−7^	4	1.3 × 10^−7^	8		
Ozone								
From NO_x_	−6.8 × 10^−7^	1	−6.9 × 10^−7^	3	−6.9 × 10^−7^	7	−1.5 × 10^−5^	10
From VOC			1.7 × 10^−6^	4	1.4 × 10^−6^	8		

Blank cells indicate values not estimated in any scenario runs. iF results reported are unitless. To calculate pollutant-specific iFs, we compared population exposures among scenarios, where each number is the difference between the scenario and the baseline scenario. For example, 1 means the corresponding iF is calculated based on the population exposure difference between the baseline scenario and scenario 1. When multiple scenarios are listed, iF was calculated based on the difference between each scenario listed and the baseline case, as well as the difference among the scenarios listed.

**Table 4:** Mortality change estimates for control scenarios by sector and pollutant.

Scenario	Sector	Pollutant controlled	Emission reductions from base–case (1,000 tons/year)	PM-related mortality change per year	Ozone-related mortality change per year	Net mortality change (PM and ozone) per year	Net mortality change per year per ton of emissions
1	Power	NO_x_	610	2,000 (200, 4,000)	−420 (−210, −630)	1,600 (350, 2,900)	2.7 × 10^−3^
2	Power	SO_2_	1,300	12,000 (1,200, 24,000)	0	12,000 (1,200, 24,000)	9.2 × 10^−3^
3	Mobile	NO_x_	83	260 (26, 520)	−60 (−30, −90)	210 (41, 380)	2.5 × 10^−3^
4	Mobile	VOC	200	380 (38, 750)	38 (19, 57)	430 (190, 680)	2.1 × 10^−3^
5	Mobile	Primary PM	6	620 (62, 1,200)	0	620 (62, 1,200)	1.0 × 10^−1^
7	Industry	NO_x_	96	300 (30, 610)	−66 (−33, −99)	250 (51, 450)	2.6 × 10^−3^
8	Industry	VOC	300	310 (31, 610)	45 (22, 67)	360 (160, 570)	1.2 × 10^−3^
9	Industry	Primary PM	110	12,000 (1,200, 24,000)	0	12,000 (1,200, 24,000)	1.1 × 10^−1^
10	Domestic	NO_x_	15	−21 (−2, −42)	−22 (−11, −33)	−44 (−59, −29)	−3.0 ×10^−3^

All values are provided to two significant figures, and sums may not add due to rounding. A positive value in the last four columns means mortality reduction (or fewer deaths), and a negative value means mortality increase (or more deaths). Values in parentheses represent plausible upper and lower bounds for pollutant-specific mortality changes and 5th and 95th percentile values from a Monte Carlo simulation for net mortality changes. Scenario 6 is not shown here because it is included as a sensitivity test. Calculating pollutant-specific population exposures and mortality changes based on this scenario would require additional modeling scenarios (e.g., two additional scenarios similar to scenarios 3 and 4, but with a reduction percentage of 50%, respectively).
